# Follistatin Protein Enhances Satellite Cell Counts in Reinnervated Muscle

**DOI:** 10.1055/s-0042-1748535

**Published:** 2022-06-21

**Authors:** Mark A. Feger, Jonathan Isaacs, Satya Mallu, Dorne Yager, Mary Shall, Gaurangkumar Patel, Omar Protzuk, Akhil S. Bokkisam

**Affiliations:** 1 Department of Orthopedic Surgery, Washington University School of Medicine, St. Louis, Missouri, United States; 2 Division of Hand Surgery, Department of Orthopaedic Surgery, Virginia Commonwealth University Medical Center, Richmond, Virginia, United States; 3 Divison of Plastic Surgery, Department of General Surgery, Virginia Commonwealth University Medical Center, Richmond, Virginia, United States; 4 Department of Physical Therapy, Virginia Commonwealth University Medical Center, Richmond, Virginia, United States

**Keywords:** peripheral nerve, anabolic, denervation atrophy, nerve repair, rodent, nerve regeneration, hypertrophy, muscle force, follistatin, progenitor cells

## Abstract

**Background** Muscle recovery following peripheral nerve repair is sup-optimal. Follistatin (FST), a potent muscle stimulant, enhances muscle size and satellite cell counts following reinnervation when administered as recombinant FST DNA via viral vectors. Local administration of recombinant FST protein, if effective, would be more clinically translatable but has yet to be investigated following muscle reinnervation.

**Objective** The aim of this study is to assess the effect of direct delivery of recombinant FST protein on muscle recovery following muscle reinnervation.

**Materials and Methods** In total, 72 Sprague-Dawley rats underwent temporary (3 or 6 months) denervation or sham denervation. After reinnervation, rats received FST protein (isoform FS-288) or sham treatment via a subcutaneous osmotic pump delivery system. Outcome measures included muscle force, muscle histomorphology, and FST protein quantification.

**Results** Follistatin treatment resulted in smaller muscles after 3 months denervation ( *p* = 0.019) and reduced force after 3 months sham denervation ( *p* < 0.001). Conversely, after 6 months of denervation, FST treatment trended toward increased force output ( *p* = 0.066). Follistatin increased satellite cell counts after denervation ( *p* < 0.001) but reduced satellite cell counts after sham denervation ( *p* = 0.037).

**Conclusion** Follistatin had mixed effects on muscle weight and force. Direct FST protein delivery enhanced satellite cell counts following reinnervation. The positive effect on the satellite cell population is intriguing and warrants further investigation.

## Introduction

Incomplete functional recovery following major peripheral nerve repair, 1 2 3 4 5 is due in part, to denervation atrophy. With loss of axonal trophic support, muscle fibers lose size, nuclei, and contractibility. 6 7 8 Though incompletely understood, satellite cells play a key role in this process as they represent a self-replicating pool of reparative cells that fuse with the atrophic cells in an attempt to preserve muscle fiber mass. 9 10 11 12 13 This pool of satellite cells is progressively depleted, resulting in irreversible muscle loss 9 14 in the absence of timely reinnervation. 14 15

Although denervation atrophy cannot be prevented from occurring following prolonged denervation, there may be strategies to compensate for residual postreinnervation muscle deficits. Augmenting muscle recovery following reinnervation has previously been attempted with nandrolone, an anabolic steroid, though this was unsuccessful. 16 This effort may have been hindered by the limited anabolic potential of nandrolone at the clinically recommended safe dosage. Pursuant of more potent anabolic agents, the myostatin-follistatin (FST) axis, could provide a more efficacious target for the enhancement of muscle function following reinnervation. Myostatin is in the TGF-β superfamily of signal transduction proteins and is a natural inhibitor of muscle growth. 17 18 Spontaneous 19 20 21 and artificially produced genetic defects 17 as well as experimental myostatin neutralization 22 all result in impressive muscle hypertrophy. Follistatin, however, a naturally occurring glycoprotein not only blocks the effects of myostatin, but also has independent muscle stimulatory 23 and pro-regenerative properties such as faster muscle healing, decreased muscle fibrosis, and enhanced satellite cell proliferation. 24

The purpose of our study was to evaluate the ability of locally administered FST protein to enhance muscle recovery following a period of temporary denervation and subsequent surgical repair. Most studies evaluating the therapeutic potential of FST have focused on systemic muscle loss such as seen in a variety of muscular dystrophies. As such, experimental FST administration has most commonly been achieved via systemic delivery of FST DNA with viral vectors. We recently demonstrated enhanced muscle mass and bolstered satellite cell pools in a companion study utilizing viral vectors expressing FST DNA. 25 However, for the focal weakness that might be seen with denervation atrophy, targeted administration of FST protein would seem more practical and clinically translatable.

## Materials and Methods

### Study Overview

**Fig. 1:**
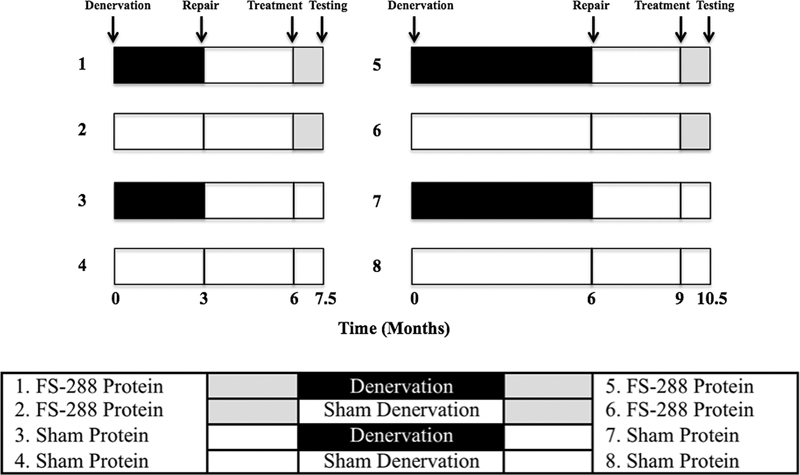
Overview of study design.

The goal of our study was to evaluate the effects of recombinant FST protein when delivered locally to reinnervated muscle following temporary denervation periods of 3 or 6 months. We utilized the same previously published methods with viral vectors that expressed FST DNA, 25 and the current investigation utilizes the same protocol to assess the effect of direct protein delivery. Eight groups of male Sprague-Dawley rats (72 rats total) were used in the study ( Fig. 1 ). Half of the rats underwent hind limb denervation (by tibial nerve transection) for 3 or 6 months before nerve repair, followed by 12 weeks of reinnervation. After 12 weeks, groups 1 and 5 were administered recombinant FST protein (isoform FS-288). Groups 3 and 7 received a representative sham treatment (vehicle only). The other half of the rats underwent sham surgeries and 3 or 6 months of matched “denervation” periods followed by a sham repair surgery. After twelve weeks, groups 2 and 6 were treated with recombinant FST protein, and groups 4 and 8 received sham treatment. Main outcome measures included muscle function, histomorphometry, and FST protein levels. The institutional animal care and use committee approved all aspects of this study.

### Surgical Manipulations

For all surgeries, general anesthesia was induced (5% isoflurane) in a closed chamber and maintained (2–3% isoflurane) via nose cone inhalation. All procedures were performed with sterile technique. The denervation protocol for both recombinant FST protein and sham treatment groups were identical. The left sciatic nerve was exposed via a standard biceps femoris semi-tendinosis muscle splitting approach, and the tibial nerve was transected just past the bifurcation. The two nerve ends were separated, buried in muscle, and held in place by a 10–0 suture to aid in identification during subsequent surgery, prevent neuroma formation, and prevent inadvertent nerve regeneration. 26 Sham denervation groups underwent sham operations in which nerves were exposed but not transected. At either 3 or 6 months, all rats underwent a second survival surgery. The left hind limb was reopened and the sciatic nerve and its divisions re-exposed. For denervated groups, the transected tibial nerve was repaired by using 1 cm of tibial nerve autograft from the contralateral leg (to avoid tension) using two or three epineural 10–0 nylon sutures per repair site.

**Fig. 2:**
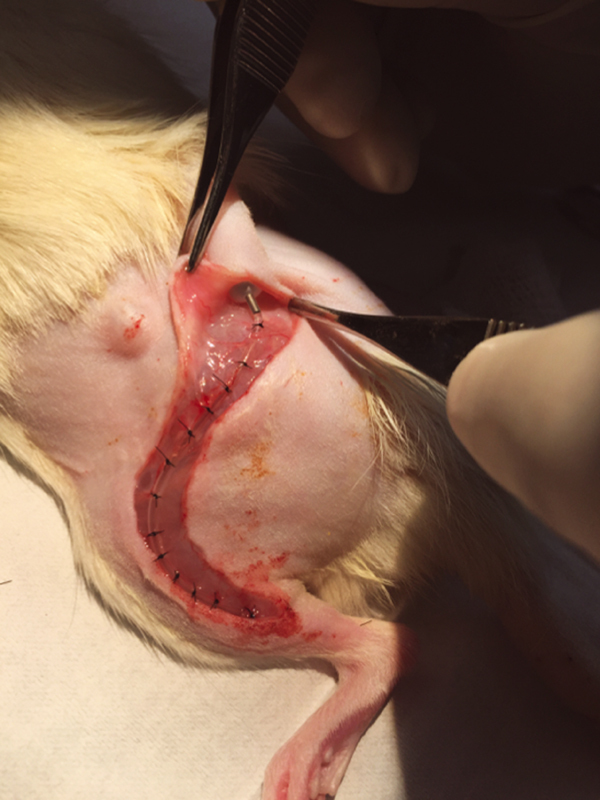
Subcutaneous osmotic pump delivery system utilized for recombinant follistatin protein and sham protein treatment groups.

After 12 weeks of nerve regeneration (to allow axons to regenerate to the muscles), all rats underwent a third survival surgery. A subcutaneous osmotic pump drug delivery system with 200 μL reservoir and delivery rate of 0.25 μL/hour ( Fig. 2 , model 2ML4 Alzet, Durect Corporation, Cupertino, California, United States) was placed in the lumbar area to administer a continuous infusion of recombinant FST protein suspended in phosphate buffered saline (PBS) or PBS only. Rats in FST treatment groups received 90 μg of commercially available recombinant FST isoform FS-288 (BioVision Incorporated, Milpitas, California, United States) suspended in 200 μL of PBS, whereas sham groups only received 200 μL of PBS.

Postoperative analgesia was achieved with subcutaneous administration of buprenorphine 0.5 mg/kg. All animals were observed for the signs of distress every 12 hours for 3 days postoperatively and then daily.

All outcome measures were recorded 1.5 months postinitiating “treatment” (FST protein or sham). At the conclusion of testing, all animals were euthanized with an intraperitoneal injection of 150 mg euthasol and were disposed of according to institutional policy.

### Muscle Function

Terminal muscle strength for all groups consisted of exposure of the tibial nerve and isolation of the gastrocnemius muscle and tendon. The hind limb was secured to a platform via placement of Kirschner wires through the femoral condyle and distal tibia. The gastrocnemius tendon was transected and coupled to a MLT500/A force transducer (AD Instruments, Inc., Colorado Springs, Colorado, United States) by using 4–0 silk suture. Strength testing was performed with a Grass stimulator (Model SD9, Astro-Med Inc., West Warwick, Rhode Island, United States) and platinum electrodes. Muscle fiber length were optimized based on the Blix curve as recommended by Shin et al, 27 and three supramaximal stimulations (5V, 1 Hz) were delivered to the sciatic nerve with 2-minute rest intervals between stimulations. Contraction strength was converted to digital data by using ADI Instruments Power Laboratory system (AD Instruments, Inc., Colorado Springs, Colorado, United States) and recorded by using a Sony VAIO laptop computer (Sony Corporation, Tokyo, Japan). At the conclusion of muscle testing, gastrocnemius muscles were harvested and weighed.

### Muscle Histomorphometric Analysis

The harvested muscles were immersed in TBS Tissue Freezing Medium (Medical Sciences, Inc., Durham, North Carolina, United States), maintaining medial and lateral orientation and in vivo length in a plastic histology mold (TedPella, Inc. Redding, California, United States). The embedded muscle was rapidly frozen by plunging into isopentane cooled in liquid nitrogen for 1 minute and stored in a freezer at −70°C. A 1-cm block was cut transversely from each muscle 5 mm distal to its origin. Serial 10-µm transverse cryostat sections were prepared from each muscle block, collected on Fisher Superfrost Plus Microscope Slides (Fisher Scientific, Suwanee, Georgia, United States) and air dried before being returned to the freezer.

Mouse monoclonal antisera was used for immunostaining of myosin heavy chains (MHC) and skeletal muscle satellite cells, including rat MHC I antibody (WB-MHs; Vector laboratories, Burlingame, California, United States), MHC 2a antibody (SC-71) (Developmental Studies Hybridoma Bank, University of Iowa, Iowa, United States), MHC 2b antibody (BF-F3; Developmental Studies Hybridoma Bank, University of Iowa), and Satellite Cell Pax7 antibody (sc-81975; Santa Cruz Biotechnology, Inc. Dallas, Texas, United States). Muscle sections were placed in 10 mM of PBS (pH = 7.5). Nonspecific binding was blocked by a 20-minute incubation period in 10% normal blocking serum. The sections were then incubated with diluted (1:50) primary antibody for 1 hour, rinsed with PBS, and incubated with diluted (1:1,000) biotinylated secondary antibody for 30 minutes. After rinsing in PBS, the sections were reacted with Vectastain Elite ABC Reagent (Vector Laboratories, Inc., Burlingame, California, United States) for 30 minutes, followed by another wash in 10 mM of PBS. A diaminobenzidine solution was used for visualization (Vector DAB kit, Vector Laboratories). The stained sections were dehydrated in ascending alcohols, cleared in xylene, and mounted in permount. An Image-ProPlus image analysis system (v. 7.0) with a Nikon Microphot-7xA Microscope and a Q Imaging digital camera (Media Cybernetics, Silver Spring, Maryland, United States), and Image-Pro v. 7.01 image analysis software were used to analyze the minimum diameter and area of muscle fibers. One hundred positive fibers were analyzed from random areas from each muscle section.

### Electrophoresis of Myosin Heavy Chains

The remainder of the gastrocnemius muscle was used for differential MHC analysis. Frozen muscles were lyophilized, minced with scissors, and homogenized with a pellet pestle in ice-cold extraction buffer (NaCl = 0.3M, Na _2_ HPO _4_ = 0.15M, EDTA = 10 mM, pH = 6.5). The solution was agitated and stirred at 4°C for 60 minutes and centrifuged (×10,000 gravity) for 10 minutes. The total protein concentrations of the supernatants were determined by using the Bio-Rad protein assay for microtiter plates (Bio-Rad Laboratories, Hercules, California, United States), based on the Bradford dye-binding procedure. The supernatants were diluted to 0.25 mg/mL in extraction buffer and stored at −70°C. MHC isoforms were separated by using a sodium dodecyl sulfate-polyacrylamide gel electrophoresis (SDS-PAGE) technique. This technique enables the separation of MHC isoforms typically expressed in rat skeletal muscles.

Gel slabs (0.75 mm thick) consisted of a 13.5-cm 8% separating gel and a 4-cm 4% stacking gel. All gels were made from the same stock solutions and all chemicals were of electrophoresis grade. A 2× Laemmli sample buffer was added to the muscle samples to yield a final protein concentration of 0.125 mg/mL. Samples were boiled for 5 minutes to denature the protein. Each lane on a gel was loaded with 20 µL of a muscle sample. Tris-glycine-SDS running buffers cooled to 4°C were used, and electrophoresis was performed by using a vertical slab gel unit (Protean II xi Cell, Bio-Rad Laboratories) run at 275V for 30 hours at 4°C.

Separating gels were silver stained by using the Silver Stain Plus Kit (Bio-Rad Laboratories). Images of silver-stained gels were obtained by using an AGFA Duoscan HiD scanner (AGFA Corporation, Ridgefield Park, New Jersey, United States). Relative proportions of MHC isoforms were determined by using Gel-Pro Analyzer (Media Cybernetics, Silver Spring, Maryland, United States), image analysis software.

### Follistatin Protein Quantification

Soluble protein was isolated from muscle tissue with a mammalian tissue lysis and extraction reagent (CelLytic, Sigma-Aldrich, Saint Louis, Missouri, United States). Muscle FST levels were measured with a human FST immunoassay kit (Quantikine ELISA Kit, R&D Systems, Minneapolis, Minnesota, United States). A total of 100 μg of protein was analyzed and prepared according to manufacturer specifications. Muscle FST concentrations were determined by comparison to a standard curve created from recombinant human FST using a Tecan Sunrise OEM Microplate Absorbance Reader.

### Statistical Analysis

The primary independent variable was treatment (FST protein or sham treatment). To provide context for any potential treatment effects, we also analyzed the effect of denervation in comparison to sham denervation within each treatment group and the difference between short (3 months) and long (6 months) denervation periods in sham denervated groups. The dependent variables were muscle force (N), muscle weight (g), muscle fiber type analysis (muscle fiber type area (µm ^2^ ), diameter (µm), and proportion), satellite cell counts, and FST levels (pg FST/mg of protein). For each dependent variable, a one-way analysis of variance (ANOVA) with LSD post hoc test was performed to assess differences between groups. The level of significance was set a priori at *p* ≤ 0.05. Data were analyzed by using Statistical Package for Social Sciences (SPSS) Version 20.0 (SPSS, Inc, Chicago, Illinois, United States).

## Results

One animal from the 3-month groups (group 1) and two animals in the 6-month groups (one rat each from groups 5 and 6) died and could not be included in the final analysis. Two additional rats from the 6-month groups (one each from groups 5 and 7) had force data excluded for technical reasons (poor muscle response during nerve stimulation), but these animals were included in other analyses.

There were significant differences ( *p* < 0.05) for all of our dependent variables between groups as determined by one-way ANOVA with the exception of the proportion of type I fibers in the 6-month groups, which was the only nonsignificant one-way ANOVA, (F [7,61] = 1.517, *p* = 0.179). The remainder of the results section includes LSD post hoc group comparisons for all other dependent variables.

### Muscle Force (N)

When holding treatment constant, denervation for 3 and 6 months resulted in reduced force when compared with matched sham denervation ( Tables 1 and 2 , *p* <0.03 for all four group comparisons). In the 3-month sham denervation groups, treatment with FST reduced force production when compared with sham treatment (FST = 0.979 ± 0.467 N vs. sham = 1.745 ± 0.596 N, *p* < 0.001). After 6 months of denervation and subsequent repair, treatment with FST trended toward increased force production when compared with sham treatment (FST = 0.630 ± 0.448 N vs. sham = 0.201 ± 0.180 N, *p* = 0.066). There were no other significant differences in force output between treatment groups.

**Table 1:** Muscle histomorphometry means, standard deviations (in parentheses), and statistical results for short (3 months) temporary denervation groups

	Muscle force (N)	Muscle weight (g)	Type I			Type IIA			Type IIB			Satellite cell count
			Area (µm ^2^ )	Diameter (µm)	Proportion (%)	Area (µm ^2^ )	Diameter (µm)	Proportion (%)	Area (µm ^2^ )	Diameter (µm)	Proportion (%)	
FST protein and denervation	0.37 (0.19)	0.64 a (0.18)	1,988.8 (803.1)	37.4 a (10.1)	20.3 (6.0)	1,371.9 a (554.4)	29.2 a (7.5)	28.7 (7.1)	1,958.8 (992.0)	37.4 (10.1)	51.0 (8.6)	157.9 a (29.0)
Sham protein and denervation	0.45 (0.25)	0.93 a (0.17)	3,222.5 (1,068.9)	48.8 a (8.5)	16.8 (3.7)	3,237.2 a (779.1)	48.5 a (6.0)	33.5 (13.5)	2,726.7 (474.2)	44.4 (3.5)	49.7 (10.6)	114.8 a (12.1)
FST protein and sham denervation	0.98 a (0.47)	2.22 (0.17)	4,379.5 (709.1)	55.1 (6.4)	16.5 (4.6)	4,417.3 (624.3)	55.9 (5.6)	43.4 (6.8)	6,404.0 (1,220.3)	66.5 (6.4)	40.4 (6.1)	134.8 a (17.0)
Sham protein and sham denervation	1.75 a (0.60)	2.42 (0.20)	4,434.2 (750.2)	57.9 (5.2)	19.4 (7.1)	4,673.8 (404.0)	58.3 (3.7)	32.9 (6.6)	5,592.9 (1,355.8)	62.6 (7.2)	47.7 (6.7)	153.0 a (16.7)

Abbreviations: FST, follistatin; N, newton; µm, micrometers.

a Denotes significant differences between treatment and matched sham treatment at *p* <0.05.

**Table 2:** Muscle histomorphometry means, standard deviations (in parentheses), and statistical results for long (6 months) temporary denervation groups

	Muscle force (N)	Muscle weight (g)	Type I			Type IIA			Type IIB			Satellite cell count
			Area (µm ^2^ )	Diameter (µm)	Proportion (%)	Area (µm ^2^ )	Diameter (µm)	Proportion (%)	Area (µm ^2^ )	Diameter (µm)	Proportion (%)	
FST protein and denervation	0.63 (0.45)	1.00 (0.53)	2,268.9 (1,410.1)	39.0 a (14.2)	9.2 (2.9)	1,942.7 (1,200.2)	35.6 (12.1)	24.9 (4.7)	2,106.1 (1,120.7)	37.7 (12.1)	65.9 (3.8)	179.1 a (40.0)
Sham protein and denervation	0.20 (0.18)	0.81 (0.33)	1,312.0 (953.2)	28.6 a (12.2)	12.1 (2.2)	1,419.9 (590.4)	29.5 (7.9)	20.5 (3.5)	1,702.4 (1,010.4)	32.2 (11.8)	67.3 (2.2)	104.0 a (12.2)
FST protein and sham denervation	1.03 (0.40)	2.33 (0.36)	4,207.3 a (1,112.0)	55.6 a (9.9)	9.9 (3.3)	3,178.6 (675.6)	45.8 (6.0)	20.9 (3.2)	4,836.4 a (1,322.8)	56.8 a (8.5)	69.2 (5.2)	147.9 (31.8)
Sham protein and sham denervation	1.29 (0.63)	2.17 (0.49)	3,130.2 a (426.9)	45.8 a (4.2)	8.2 (4.3)	2,419.1 (435.5)	39.5 (4.0)	24.1 (3.3)	2,666.8 a (326.6)	42.2 a (2.9)	67.7 (2.9)	137.5 (8.8)

Abbreviations: FST, follistatin; N, newton; µm, micrometers.

a Denotes significant differences between treatment and matched sham treatment at *p* <0.05.

### Muscle Weight (g)

When holding treatment constant, denervation for 3 and 6 months resulted in lower muscle weights when compared with sham denervation groups ( Tables 1 and 2 , *p* <0.001 for all four group comparisons). Following 3 months of denervation and repair, treatment with FST resulted in lower muscle weights when compared with sham (FST = 0.636 ± 0.179 g vs. sham = 0.927 ± 0.167 g, *p* = 0.019). There were no other significant differences in muscle weight between treatment groups.

### Muscle Histology

#### Muscle Fiber Area, Diameter, and Proportion

When holding treatment constant, denervation for 3 and 6 months resulted in significantly smaller fiber areas and diameters of type I, type IIA, and type IIB fibers when compared with sham denervation groups ( Tables 1 and 2 , *p* <0.05 for 21 of 24 group comparisons) with no consistent differences in the proportion of fiber type ( *p* > 0.05 for 8 of 10 group comparisons). Following 3 months of denervation, FST treatment did result in an increased proportion of type IIB fibers (denervation = 51.0 ± 8.6% vs. sham denervation = 40.4 ± 6.1%, *p* = 0.009) and a decreased proportion of type IIA (denervation = 28.7 ± 7.1% vs. sham denervation = 43.4 ± 6.8%, *p* < 0.001) when compared with sham denervation. However, in FST protein treatment groups, 6 months of denervation resulted in an increased proportion of type IIA fibers when compared with sham denervation (denervation = 24.9 ± 4.7% vs. sham denervation = 20.9 ± 3.2%, *p* = 0.041).

Following 3 months of denervation, treatment with FST decreased the diameter of type 1 fibers (FST = 37.4 ± 10.1 µm vs. sham = 48.8 ± 8.5 µm, *p* = 0.003) and the area (FST = 1,371.9 ± 554.4 µm2 vs. sham = 3,237.2 ± 779.1 µm ^2^ , *p* < 0.001) and diameter (FST = 29.2 ± 7.5 µm vs. sham = 48.5 ± 6.0 µm, *p* < 0.001) of type IIA fibers when compared with sham treatment.

Following 6 months of denervation and repair, treatment with FST resulted in larger diameters of type I muscle fibers (FST = 39.0 ± 14.2 µm vs. sham = 28.6 ± 12.2 µm, *p* = 0.035) and trended toward larger type I fiber areas ( *p* = 0.054). In 6-month sham denervation groups, treatment with FST resulted in larger areas and diameters of type I ( *p* = 0.030 and 0.046, respectively) and type IIB ( *p* = 0.001 and 0.009, respectively) fiber types with a trend toward larger type IIA fiber areas ( *p* = 0.051) when compared with sham treatment. There were no other significant differences in muscle fiber type area, diameter, or proportion.

#### Satellite Cell Counts

In sham treatment groups, 3 months of denervation resulted in lower satellite cell counts when compared with sham denervation ( Table 1 , *p* < 0.001). However, in groups treated with FST, 3 months of temporary denervation resulted in significantly higher satellite cell counts than sham denervation groups that were also treated with FST ( *p* = 0.002). In the 3-month denervation groups, treatment with FST (FST = 157.9 ± 29.0 vs. sham = 114.8 ± 12.1, *p* < 0.001) increased satellite cell counts compared with sham treatment. However, in 3-month sham denervation groups, treatment with FST protein decreased (FST = 134.8 ± 17.0 vs. sham = 153.0 ± 16.7, *p* = 0.037) satellite cell counts.

In sham treatment groups, 6 months of denervation resulted in lower satellite cell counts when compared with sham denervation ( Table 2 , *p* = 0.045) However, in groups treated with FST, 6 months of denervation resulted in significantly higher satellite cell counts than sham denervation groups that were also treated with FST ( *p* = 0.012). Following 6 months of denervation, treatment with FST (FST = 179.1 ± 40.0 vs. sham = 104.0 ± 12.2, *p* < 0.001) significantly increased satellite cell counts compared with sham treatment. There were no other significant differences between treatment groups for the number of satellite cells.

### Follistatin Protein Quantification (pg Follistatin/mg of Protein)

In 3-month sham treatment groups, there were no differences in the level of FST protein between temporary denervation groups and their respective sham denervation groups ( Table 3 , *p* > 0.05). The FST level in all 6-month groups were below the detection threshold and thus were unable to be quantified or utilized for group comparisons. There were no other significant differences in FST protein concentration between treatment groups.

**Table 3:** Follistatin level means, standard deviations (in parentheses), and statistical results for short (3 months, left) and long (6 months, right) temporary denervation groups

3-month groups	Follistatin protein level (pg follistatin/mg of protein)	6-month groups	Follistatin protein level (pg follistatin/mg of protein)
FST protein and denervation	2,678.8 (2,183.6)	FST protein and denervation	Not detectable
Sham protein and denervation	2,649.8 (608.7)	Sham protein and denervation	Not detectable
FST protein and sham denervation	2,282.0 (1,516.9)	FST protein and sham denervation	Not detectable
Sham protein and sham denervation	3,301.4 (715.3)	Sham protein and sham denervation	Not detectable

Abbreviations: FST, follistatin, pg, picograms; mg, milligrams.

a Denotes significant differences between treatment and matched sham treatment at *p* <0.05.

### Effect of Increased Length of Denervation and Sham Denervation in Sham Groups

**Fig. 3:**
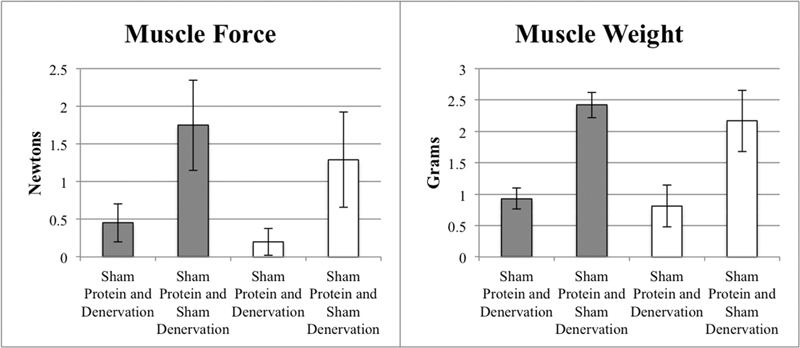
Effect of denervation versus sham denervation (comparisons within same color) and effect of short (3 months, gray) and long (6 months, white) denervation (comparisons between colors) on muscle force (left) and muscle weight (right) in sham treatment groups.

**Fig. 4:**
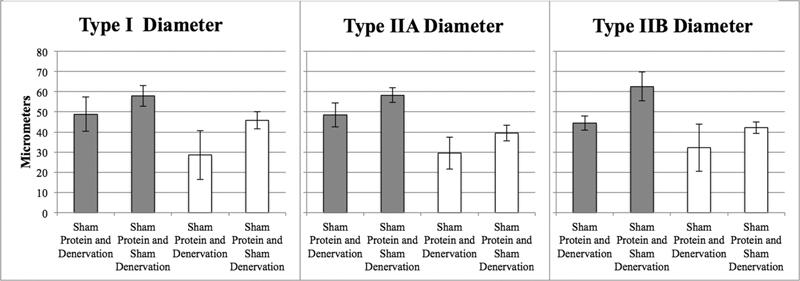
Effect of denervation versus sham denervation (comparisons within same color) and effect of short (3 months) and long (6 months) denervation (comparisons between colors) on Type I, IIA, and IIB fiber diameters in 3 (gray) and 6 months (white) sham treatment groups.

Increasing the temporary denervation period from 3 to 6 months had no effect on muscle weight or force production ( Fig. 3 ). Increasing the sham denervation period from 3 to 6 months had no effect on muscle weight or force production ( Fig. 3 ). Three additional months of temporary denervation and sham denervation consistently resulted in smaller individual type 2B, type 2A, and type I muscle fiber areas and diameters ( Fig. 4 , *p* < 0.05 in 11 of 12 group comparisons) with a shift from type 2B to type 2A and type I fiber types ( *p* < 0.05 in four of six analyses). There was no change in satellite cell counts between short and long denervation periods.

## Discussion

Therapeutic administration of FST has been achieved through local and systemic delivery of FST protein 28 and DNA. 29 We previously demonstrated improved muscle mass, increased type IIB muscle fiber size, and increased satellite cell counts in an identical study with the administration of FST DNA via viral vectors. 25 In contrast to transgenetic manipulation, however, the therapeutic potential of direct FST protein administration has received scant attention. Injecting spinal muscular atrophy mice with recombinant FST increased muscle mass, coordination, and life span. Of the several dosing regimens, 0.75 mg/kg twice per day was most effective. 28 Likewise, local injections into cardiotoxin damaged rodent muscle with FS-288 protein (0.5 mg/kg/day) improved muscle regeneration (9% gain in muscle mass). 30

Our treatment protocol aimed to provide comparable dosages (1.5 mg/kg/day) locally with an osmotic implantable pump and catheter delivery system. Dosing was based upon estimated target muscle weight as opposed to total animal weight. Unpublished pilot data from our laboratory unambiguously confirmed an anabolic effect with this strategy when healthy muscles were assessed immediately after a 30-day treatment. However, the effects of this treatment approach after repeated surgical manipulations in real and sham denervation groups has proven more difficult to interpret. Paradoxically, FST treatment after short temporary denervation resulted in smaller muscles driven by reductions in type I and type IIA fiber size. Gangopadhyay noted an analogous decrease in type I and IIA fiber though with an increase in type IIB fibers following systemic administration of FS-288. 31 Follistatin treatment in the 3-month sham denervation group was also associated with decreased muscle force, but larger type I fibers and a trend toward increased muscle force after 6 months of temporary denervation.

When analyzing the effect of denervation alone, denervation predictably resulted in reduced satellite cell counts (in sham groups), which is consistent with existing literature. 32 When analyzing the effect of FST treatment alone, FST decreased satellite cell counts in sham groups, whereas it bolstered the satellite cell pool in denervation groups. To our knowledge, this is the first study to demonstrate this unique differential effect of FST and denervation on the satellite cell pool depending on environmental conditions of the muscle though we are unable to explain the mechanism behind this relationship. Likewise, our prior investigation demonstrated that recombinant FST DNA also enhances satellite cell counts after denervation and repair. 25 In the current investigation, there did seem to be a correlation with satellite cell counts and force generation. In groups in which the satellite cell counts were diminished, (3-month sham denervation) force output was reduced and conversely in groups with bolstered satellite cell counts (6-month denervation) force output was enhanced. This may suggest that further investigation into this relationship between satellite cell counts and motor function is warranted.

Follistatin treatment was initiated 6 weeks prior to testing and was delivered over 4 weeks. The “withdrawal” of exogenous FST after reservoir depletion may have resulted in a ratio of endogenous FST to myostatin that favored a catabolic process during the remaining 2 weeks. This effect was not evident in our pilot analysis when outcomes were analyzed immediately after the conclusion of protein delivery or in our companion study with FST DNA that was able to maintain elevated FST protein levels throughout the conclusion of testing. 25 Follistatin to myostatin ratios have been shown to correlate with denervation atrophy and regeneration, 33 and myostatin is inhibited by FST in a direct concentration-dependent manner. 34 It is conceivable that a temporary spike in FST triggered an upregulation of myostatin, which is known to cause muscle atrophy 35 and any treatment effect may have peaked at the end of the FST treatment and regressed prior to testing. Why this effect was not observed in longer denervation groups may reflect a more profound degradation of muscle fibers (which secrete myostatin) so that the remaining tissue was not capable of the same response. Regardless, muscle atrophy and hypertrophy are complex processes that encompass multiple growth factors and trying to explain our findings in terms of only myostatin and FST levels is likely a gross oversimplification.

There were no detectable levels of FST protein in treated and untreated muscles in the 6-month groups. Unlike genetically based FST treatment, 25 focal FST protein delivery would only elevate local levels of FST during the course of administration and analysis was completed 2 weeks after the reservoir was depleted. Any FST being quantified would be endogenously produced, and the failure to demonstrate FST even in untreated groups implies that local production of FST is impaired after 6 months of repeated surgical manipulations, temporary denervation, catheter irritation, and/or sedentary behavior. When compared with our prior investigation, 25 this information is important as it demonstrates the need for a sustained presence of FST to maintain muscle mass and function after reinnervation. Armand et al detected increased FST mRNA in myotubes but only in late stages of regeneration 36 which may not have been applicable to our study as demonstrated by the profound atrophy still present in the untreated muscles after prolonged denervation.

It is also worth considering that treated muscles may have gradually been getting stronger, and a later testing point, a longer treatment period, or different dosing strategy/method may have all revealed a more profound treatment effect. This theory is supported by the apparent positive influence of FST on the 6-month denervation groups where the magnitude of treatment effect would have been expected to be the greatest. When comparing and contrasting the results of FST protein versus FST DNA delivery 25 in two analogous studies, FST administration regardless of delivery method enhances satellite cell counts but muscle size and function require persistent elevations in FST protein levels. The consistent positive effect on the satellite cell population in temporarily denervated muscle after both FST protein and FST DNA 25 treatment is intriguing and warrants further study.

### Limitations

The administration of FST had a positive effect on satellite cells, but this study is not without limitations. The timing of FST delivery with regards to the denervation/reinnervation cycle, the local dosing regimen, the size of the autograft used for repair, and the timing of post-treatment data acquisition in the current study were variables that were chosen based upon the best available preliminary evidence but are areas that should be explored in future investigations. Additionally, given the preliminary nature of this study we are unable to comment on how these variables in a rodent model would translate into larger mammal models/humans. Furthermore, animal mass and activity levels were not utilized in the current study secondary to us by using the contralateral tibial nerve autograft for repair, which would have disproportionally affected these outcome measures. Lastly, the sedentary rodent housing environment did not allow us to assess the ability of FST to enhance muscle function under progressive loading conditions that would be encountered in clinical settings.

## References

[1] Sakellarides H A follow-up study of 172 peripheral nerve injuries in the upper extremity in civilians J Bone Joint Surg Am 1962 44-A 140 148 14038909

[2] Jongen S J Van Twisk R Results of primary repair of ulnar and median nerve injuries at the wrist: an evaluation of sensibility and motor recovery Neth J Surg 1988 40 03 86 89 3405445

[3] Roganovic Z Missile-caused median nerve injuries: results of 81 repairs Surg Neurol 2005 63 05 410 418, discussion 418–419 15883059 10.1016/j.surneu.2004.08.007

[4] Secer H I Daneyemez M Gonul E Izci Y Surgical repair of ulnar nerve lesions caused by gunshot and shrapnel: results in 407 lesions J Neurosurg 2007 107 04 776 783 17937222 10.3171/JNS-07/10/0776

[5] Ruijs A C Jaquet J B Kalmijn S Giele H Hovius S E Median and ulnar nerve injuries: a meta-analysis of predictors of motor and sensory recovery after modern microsurgical nerve repair Plast Reconstr Surg 2005 116 02 484 494, discussion 495–496 16079678 10.1097/01.prs.0000172896.86594.07

[6] Fu S Y Gordon T Contributing factors to poor functional recovery after delayed nerve repair: prolonged denervation J Neurosci 1995 15(5 Pt 2):3886 3895 7751953 10.1523/JNEUROSCI.15-05-03886.1995 PMC6578254

[7] Lien S C Cederna P S Kuzon W M Jr Optimizing skeletal muscle reinnervation with nerve transfer Hand Clin 2008 24 04 445 454, vii vii. 18928892 10.1016/j.hcl.2008.08.001

[8] Cederna P S Youssef M K Asato H Urbanchek M G Kuzon W M Jr Skeletal muscle reinnervation by reduced axonal numbers results in whole muscle force deficits Plast Reconstr Surg 2000 105 06 2003 2009, discussion 2010–2011 10839398 10.1097/00006534-200005000-00014

[9] Anzil A P Wernig A Muscle fibre loss and reinnervation after long-term denervation J Neurocytol 1989 18 06 833 845 2621479 10.1007/BF01187235

[10] Ontell M Muscle satellite cells: a validated technique for light microscopic identification and a quantitative study of changes in their population following denervation Anat Rec 1974 178 02 211 227 4131292 10.1002/ar.1091780206

[11] Schultz E Changes in the satellite cells of growing muscle following denervation Anat Rec 1978 190 02 299 311 629408 10.1002/ar.1091900212

[12] Moss F P Leblond C P Satellite cells as the source of nuclei in muscles of growing rats Anat Rec 1971 170 04 421 435 5118594 10.1002/ar.1091700405

[13] Hill M Wernig A Goldspink G Muscle satellite (stem) cell activation during local tissue injury and repair J Anat 2003 203 01 89 99 12892408 10.1046/j.1469-7580.2003.00195.x PMC1571137

[14] Kobayashi J Mackinnon S E Watanabe O The effect of duration of muscle denervation on functional recovery in the rat model Muscle Nerve 1997 20 07 858 866 9179158 10.1002/(sici)1097-4598(199707)20:7<858::aid-mus10>3.0.co;2-o

[15] Aydin M A Mackinnon S E Gu X M Kobayashi J Kuzon W M Jr Force deficits in skeletal muscle after delayed reinnervation Plast Reconstr Surg 2004 113 06 1712 1718 15114133 10.1097/01.prs.0000118049.93654.ca

[16] Isaacs J Feher J Shall M Effects of nandrolone on recovery after neurotization of chronically denervated muscle in a rat model J Neurosurg 2013 119 04 914 923 23829817 10.3171/2013.5.JNS121837

[17] McPherron A C Lawler A M Lee S J Regulation of skeletal muscle mass in mice by a new TGF-beta superfamily member Nature 1997 387(6628):83 90 9139826 10.1038/387083a0

[18] Langley B Thomas M Bishop A Sharma M Gilmour S Kambadur R Myostatin inhibits myoblast differentiation by down-regulating MyoD expression J Biol Chem 2002 277 51 49831 49840 12244043 10.1074/jbc.M204291200

[19] McPherron A C Lee S J Double muscling in cattle due to mutations in the myostatin gene Proc Natl Acad Sci U S A 1997 94 23 12457 12461 9356471 10.1073/pnas.94.23.12457 PMC24998

[20] Kambadur R Sharma M Smith T P Bass J J Mutations in myostatin (GDF8) in double-muscled Belgian Blue and Piedmontese cattle Genome Res 1997 7 09 910 916 9314496 10.1101/gr.7.9.910

[21] Grobet L Martin L J Poncelet D A deletion in the bovine myostatin gene causes the double-muscled phenotype in cattle Nat Genet 1997 17 01 71 74 9288100 10.1038/ng0997-71

[22] Bogdanovich S Krag T O Barton E R Functional improvement of dystrophic muscle by myostatin blockade Nature 2002 420(6914):418 421 12459784 10.1038/nature01154

[23] Lee S J Quadrupling muscle mass in mice by targeting TGF-beta signaling pathways PLoS One 2007 2 08 e789 17726519 10.1371/journal.pone.0000789 PMC1949143

[24] Zhu J Li Y Lu A Follistatin improves skeletal muscle healing after injury and disease through an interaction with muscle regeneration, angiogenesis, and fibrosis Am J Pathol 2011 179 02 915 930 21689628 10.1016/j.ajpath.2011.04.008 PMC3157209

[25] Isaacs J Feger M A Mallu S Viral vector delivery of follistatin enhances recovery of reinnervated muscle Muscle Nerve 2019 60 04 474 483Epub20190801. 31365129 10.1002/mus.26653

[26] Isaacs J Loveland K Mallu S Adams S Wodicka R The use of anabolic steroids as a strategy in reversing denervation atrophy after delayed nerve repair Hand (N Y) 2011 6 02 142 148 22654697 10.1007/s11552-011-9331-y PMC3092896

[27] Shin R H Vathana T Giessler G A Friedrich P F Bishop A T Shin A Y Isometric tetanic force measurement method of the tibialis anterior in the rat Microsurgery 2008 28 06 452 457 18623151 10.1002/micr.20520

[28] Rose F F Jr Mattis V B Rindt H Lorson C L Delivery of recombinant follistatin lessens disease severity in a mouse model of spinal muscular atrophy Hum Mol Genet 2009 18 06 997 1005 19074460 10.1093/hmg/ddn426 PMC2649020

[29] Rodino-Klapac L R Haidet A M Kota J Handy C Kaspar B K Mendell J R Inhibition of myostatin with emphasis on follistatin as a therapy for muscle disease Muscle Nerve 2009 39 03 283 296 19208403 10.1002/mus.21244 PMC2717722

[30] Yaden B C Croy J E Wang Y Follistatin: a novel therapeutic for the improvement of muscle regeneration J Pharmacol Exp Ther 2014 349 02 355 371 24627466 10.1124/jpet.113.211169

[31] Gangopadhyay S S Systemic administration of follistatin288 increases muscle mass and reduces fat accumulation in mice Sci Rep 2013 3 2441 23942549 10.1038/srep02441 PMC3743061

[32] Viguie C A Lu D X Huang S K Rengen H Carlson B M Quantitative study of the effects of long-term denervation on the extensor digitorum longus muscle of the rat Anat Rec 1997 248 03 346 354 9214552 10.1002/(SICI)1097-0185(199707)248:3<346::AID-AR7>3.0.CO;2-N

[33] Wu R H Wang P Yang L Li Y Liu Y Liu M A potential indicator of denervated muscle atrophy: the ratio of myostatin to follistatin in peripheral blood Genet Mol Res 2011 10 04 3914 3923 22033906 10.4238/2011.October.21.7

[34] Amthor H Nicholas G McKinnell I Follistatin complexes myostatin and antagonises Myostatin-mediated inhibition of myogenesis Dev Biol 2004 270 01 19 30 15136138 10.1016/j.ydbio.2004.01.046

[35] Zimmers T A Davies M V Koniaris L G Induction of cachexia in mice by systemically administered myostatin Science 2002 296(5572):1486 1488 12029139 10.1126/science.1069525

[36] Armand A S Della Gaspera B Launay T Charbonnier F Gallien C L Chanoine C Expression and neural control of follistatin versus myostatin genes during regeneration of mouse soleus Dev Dyn 2003 227 02 256 265 12761853 10.1002/dvdy.10306

